# *RAS* Mutation Conversion in Bevacizumab-Treated Metastatic Colorectal Cancer Patients: A Liquid Biopsy Based Study

**DOI:** 10.3390/cancers14030802

**Published:** 2022-02-04

**Authors:** Chiara Nicolazzo, Francesca Belardinilli, Annarita Vestri, Valentina Magri, Gianluigi De Renzi, Michela De Meo, Salvatore Caponnetto, Federica Di Nicolantonio, Enrico Cortesi, Giuseppe Giannini, Paola Gazzaniga

**Affiliations:** 1 Department of Molecular Medicine, Sapienza University of Rome, 00185 Roma, Italy; chiara.nicolazzo@uniroma1.it (C.N.); francesca.belardinilli@uniroma1.it (F.B.); gianluigi.derenzi@uniroma1.it (G.D.R.); demeo.1461110@studenti.uniroma1.it (M.D.M.); giuseppe.giannini@uniroma1.it (G.G.); 2 Department of Public Health and Infectious Disease, Sapienza University of Rome, 00185 Roma, Italy; annarita.vestri@uniroma1.it; 3 Department of Radiology, Oncology and Pathology, Sapienza University of Rome, 00185 Roma, Italy; valentina.magri@uniroma1.it (V.M.); salvo.caponnetto@uniroma1.it (S.C.); enrico.cortesi@uniroma1.it (E.C.); 4 Department of Oncology, University of Turin, 10124 Torino, Italy; federica.dinicolantonio@unito.it; 5 Candiolo Cancer Institute, FPO-IRCCS, 10060 Candiolo, Italy

**Keywords:** liquid biopsy, bevacizumab, *RAS* conversion, colorectal cancer

## Abstract

**Simple Summary:**

Recent evidence has been provided that the clonal evolution of mutant *RAS* colorectal tumors may lead to the negative selection of mutant *RAS* clones, with the appearance of a time window characterized by the disappearance of *RAS* mutant clones in plasma. We demonstrate here for the first time that the use of bevacizumab in the first-line treatment is the most significant factor for *RAS* conversion from mutant to wild type in plasma. The frequent appearance of this “*RAS* wild-type * window” in patients treated with a first line treatment containing bevacizumab could possibly present them as candidates for second line treatment with anti-EGFR monoclonal antibodies, which are otherwise precluded.

**Abstract:**

Liquid biopsies have shown that, in *RAS* mutant colorectal cancer, the conversion to *RAS* wild-type * status during the course of the disease is a frequent event, supporting the concept that the evolutionary landscape of colorectal cancer can lead to an unexpected negative selection of RAS mutant clones. The aim of the present study was to clarify whether the negative selection of *RAS* mutation in plasma might be drug-dependent. For this purpose, we used liquid biopsy to compare the rate of conversion from *RAS* mutant to *RAS* wild-type * in two groups of originally *RAS* mutant mCRC patients: the first treated with chemotherapy alone, while the second was treated with chemotherapy combined with bevacizumab. Serial liquid biopsies were performed at 3 months (T1), 6 months (T2), 9 months (T3), and 12 months (T4) after starting first line treatments. We found that the only independent variable significantly associated to *RAS* status conversion was the use of bevacizumab. *RAS* conversion was not found associated to tumor burden reduction, although bevacizumab-treated patients who converted to *RAS* wild-type * had a significantly longer PFS compared to patients who remained *RAS* mutant. The appearance of a “*RAS* wild-type * window”, mainly in bevacizumab-treated patients, might present them as candidates for second line treatment with anti-EGFR, which was otherwise precluded.

## 1. Introduction

Activating oncogenic mutations in *KRAS* and *NRAS* (*RAS*) are common in metastatic colorectal cancer (mCRC), resulting in the constitutive activation of the Ras/Raf/MEK/ERK pathway [1]. Since driver mutations in the *RAS* gene family lead to a detrimental effect from EGFR-directed therapies, current clinical guidelines for *RAS* mutant mCRC recommend chemotherapy with the addition of anti-vascular endothelial growth factor (anti-VEGF) agents (i.e., bevacizumab, aflibercept, and ramucirumab) [2]. Among those, bevacizumab, a recombinant, humanized monoclonal antibody (MoAb) that targets VEGF, is widely used in the first-line treatment of *RAS* mutant mCRC. Bevacizumab induces a consistent improvement in progression free survival (PFS) compared to chemotherapy (CT) alone in first-line treatment (median PFS: 9–10 months), so its use is standard for most patients with no recognized contraindication [3]. Recent studies performed through liquid biopsy have provided evidence that the clonal evolution of *RAS* mutant CRC may lead to the negative selection of *RAS* mutant clones, with the appearance of a time window characterized by a *RAS* wild- type disease in plasma [4,5,6,7,8]. Some studies are currently investigating the efficacy of anti-EGFR therapy in patients with initially *RAS* mutant mCRC, who convert to *RAS* wild-type * in plasma with disease progression [9,10,11]. The phenomenon of *RAS* mutation conversion was originally described in hematological malignancies, mainly in childhood leukaemias, with *RAS* mutations being present at diagnosis but often lost at relapse, supporting the hypothesis of a negative selection of oncogenic drivers [12]. Although an increasing body of evidence suggests that in colorectal cancer the conversion from *RAS* mutant to *RAS* wild-type * status is a frequent event, whether this switch might be due to a specific treatment has not been clarified to date. Specifically, the selective pressure induced by anti-angiogenic treatments in *RAS* mutant mCRC patients is an unanswered question. The aim of the present study was to clarify whether *RAS* mutation loss in colorectal cancer might be a drug-dependent phenomenon. For this purpose, we compared the rate of conversion from *RAS* mutant to *RAS* wild-type * in plasma in two groups of originally *RAS* mutant mCRC patients: the first treated with chemotherapy alone, while the second was treated with chemotherapy combined with bevacizumab.

## 2. Materials and Methods

### 2.1. Patients and Samples Collection

A total of 72 patients with unresectable *RAS* mCRC, with concordance of RAS mutational status between baseline plasma ctDNA and primary tumor tissue, were enrolled between 2018 and 2020 before starting first-line treatment. Inclusion criteria were: males or females; age > 18 years; evidence of *RAS/BRAF* mutations concordant in primary tumor tissue and plasma samples at the time of diagnosis (T0); no previous lines of treatment received; ECOG performance status ≤2; and signed informed consent. Formalin-fixed and paraffin-embedded tissue sections from primary tumors were examined by next-generation sequencing (NGS) according to standard procedures. Blood samples for ctDNA analysis were serially collected at 3 months after starting first line treatment (T1), and then after 6 months (T2), 9 months (T3), and 12 months (T4). Treatment response evaluation according to RECIST 1.1 was performed every 9 weeks by thoracic, abdominal, and pelvic computed tomography (CT)-scan. Blood draws were performed after obtaining informed consent. Authorization to perform liquid biopsies was released by the Regional Ethical Committee (No.:179/16), and the study was conducted in accordance with the Declaration of Helsinki. Plasma samples were obtained by centrifugation of 6 mL of blood at 1500 rpm for 10 min, followed by removal of plasma, which was further centrifuged at 13,000 rpm for 1 min. Plasma samples were stored at −80 °C until use.

### 2.2. RAS Mutational Analysis in Tissue and Plasma Samples

*RAS* mutational status in tissue samples was assessed using the Oncomine™ Colon cell-free DNA Assay (Thermo Fisher Scientific). *RAS* mutation detection in plasma samples was performed through Idylla™(Biocartis). The Idylla™ ct*KRAS* Mutation Test is an in vitro diagnostic test for the qualitative detection of 21 mutations in codons 12, 13, 59, 61, 117, and 146 of the *KRAS* gene. The Idylla™ ct*NRAS*-*BRAF* Mutation Test is an in vitro diagnostic test for the qualitative detection of mutations in codons 12, 13, 59, 61, 117, and 146 of the *NRAS* gene and codon 600 of the *BRAF* gene. The overall agreement between the Idylla™ ct*KRAS* and ct*NRAS-BRAF* mutation test as compared to standard of care (SOC) tissue testing is 78.9% [13]. Only patients with concordance of RAS mutational status between baseline plasma ctDNA and tumor tissue were included. Plasma samples which resulted in *RAS/BRAF* wild-type * were further analyzed through methylation test or NGS in order to confirm the presence of ctDNA, as previously described [8]. For both methylation and NGS analysis, ctDNA was extracted from 1 mL and 4 mL of plasma, respectively, using Maxwell 16 system (Promega) according to the manufacturer’s instructions. ctDNA (20 microliters) were subjected to bisulfite conversion using the EZ DNA methylation Gold kit (Zymo Research), with final elution in 40 µL. Bisulfite converted ctDNA was assessed for the methylation status of five genes (*EYA4, GRIA4, ITGA4, MAP3K14-AS1,* and *MSC*), as previously described [14]. NGS analysis was performed using the Oncomine™ Colon cell-free DNA Assay (Thermo Fisher Scientific), containing a single primer pool to amplify hotspots and targeted regions of fourteen genes): *AKT1, BRAF, CTNNB1, EGFR, ERBB2, FBXW7, GNAS, KRAS, MAP2K1, NRAS, PIK3CA, SMAD4, TP53,* and *APC*, which frequently mutated in gastro-intestinal cancers, with a limit of detection (LOD) down to 0.1%. Twenty (20) ng of cfDNA input or a maximum volume of 13 µL per sample were used for libraries preparation, according to the manufacturer’s instructions. Templated spheres were prepared using 100 pM of each library by using the Ion One Touch 2.0 machine (Thermo Fisher Scientific, Waltham, MA, USA). Template-positive spheres were loaded into Ion chip 318 and sequenced by IT-PGM machine (Thermo Fisher Scientific, Waltham, MA, USA). Sequencing data were analyzed with the Ion Torrent Suite Software (Thermo Fisher Scientific, http://github.com/iontorrent/TS, accessed on 2 February 2022) using Coverage Analysis, Molecular Coverage Analysis, and Variant Caller plugins, and with Ion Reporter Software using the workflow Oncomine Colon Liquid Biopsy-w1.6, according to company’s recommendations. Variants were verified using the IGV visualization tool (http://www.broadinstitute.org/igv/, accessed on 2 February 2022).

### 2.3. Statistical Analysis

A descriptive analysis was performed, continuous variables were summarized using means and standard deviation, or median and interquartile range, according to each variable’s distribution; categorical variables were reported using counts and percentages. We compared the rate of conversion, from *RAS* to *RAS* wild-type * in plasma in two groups of originally *RAS* mutated mCRC patients using Fisher’s exact test; to evaluate the differences between age, we used the Mann–Whitney U test. We performed a logistic regression using *RAS* mutation conversion (yes/no) as the dependent variable. The covariates were sex, age, tumor sidedness, type of *RAS* mutation, and bevacizumab use.

A Kaplan–Meier was performed to describe the progression free survival (PFS). The log-rank test was used to compare the PFS respect the rate of conversion. All tests were two-tailed, and the level of significance was set at α < 0.05. All analysis were performed by STATA v.16.

## 3. Results

### 3.1. Study Population

The cohort included 72 patients with a primary diagnosis of mCRC, with evidence of *RAS/BRAF* mutations in both primary tumor tissue and plasma samples collected before starting first line treatments. There were 46 males and 26 females; median age at diagnosis was 67 years (range: 44–88). The number of patients with a primary tumor on the left side was 48, of which 20 were located in the rectum. Patients received first-line chemotherapy with FOLFIRI, FOLFOX, FOLFOXIRI, or 5-Fluoruracil (5-FU) with (50 patients) or without (22 patients) bevacizumab. The Response Evaluation Criteria in Solid Tumors (RECIST) were used to establish progression of the disease. The Kaplan–Meier estimate of the overall median PFS was 9.4 months (95% CI 8.7–10.1). The patient’s characteristics are shown in Table 1.

### 3.2. Tracking RAS/BRAF Mutations in Plasma Samples

Serial liquid biopsies were performed at 3 months (T1), 6 months (T2), 9 months (T3), and 12 months (T4) after starting first line treatments. In the whole population, 29 patients (40%) did not change *RAS* mutational status in the follow up, while *RAS* conversions were observed in 43 cases (60%). In the univariate analysis, we found a statistical difference between the median age of the 43 *RAS*-converting cases, compared to the 29 *RAS*-stable cases (*p* = 0.044). No difference was found between patients with and without *RAS* status conversion with respect to sex (*p* = 0.618) and to tumor sidedness (*p* = 0.612). At the first disappearance of *RAS* mutation in plasma, samples were further analyzed for colon cancer-specific methylation signature or through NGS, in order to confirm the presence of DNA of tumor origin in the circulation. In 36 out of the 43 cases who converted to wild-type * *RAS* in plasma, ctDNA presence was confirmed through NGS analysis (27 cases) and methylation test (9 cases); in the remaining 7 cases, the amount of plasma samples were not sufficient to perform further analysis. Timing of *RAS* conversion varied from 3 to 12 months according to patients, independent on the type of *RAS* mutation originally detected at baseline. In Table 2, the characteristics of 43 patients who converted to *RAS* wild-type * in plasma (type of *RAS* mutation found at baseline, timing of *RAS* conversion, and the results of the test performed to confirm ctDNA presence) are illustrated.

*RAS* mutations became undetectable in 16 patients (37%) after 3 months (T1), while conversion was observed at T2, T3, and T4 in 14 (32%), 9 (21%), and 4 (10%) patients, respectively. After the first *RAS* conversion, in 38/43 patients (88%), *RAS* wild-type * persisted for all the following timepoints, while 5 patients switched to *RAS* mutant, 2 of them showing the original *RAS* mutation and 3 a novel one, at T4 (Table 3). No relationship was found between the occurrence of *RAS* conversion and tumor response. Therefore, statistical analysis in respect to an association between RAS conversion and tumor response according to RECIST criteria was not significant (*p* = 0.8). Specifically, 12 patients (30%) had progressive disease (PD) at the time of *RAS* conversion, while 11 (26%), 18 (42%), and 2 (5%) had stable disease (SD), partial response (PR), and complete response (CR), respectively. Among the 31 patients who were not in PD at the time of *RAS* conversion, 17(55%) maintained *RAS* wild-type status in plasma until PD (Table 3).

**Figure 1:**
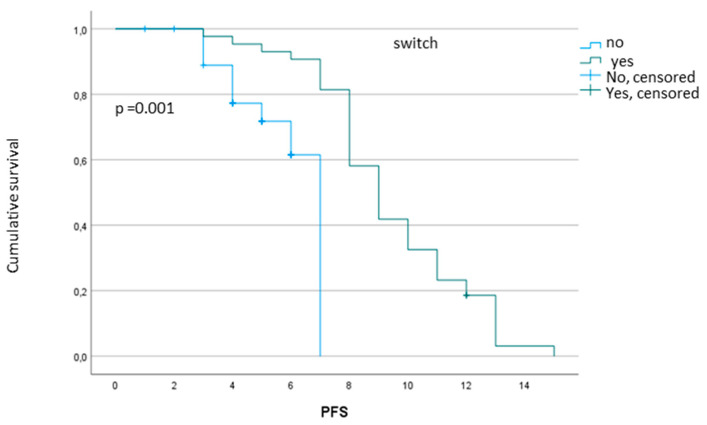
Difference in PFS between bevacizumab-treated patients who switched to *RAS* wild-type in plasma vs. patients who remained *RAS* mutant.

We then analyzed the impact of treatment regimen (CT alone vs. CT plus bevacizumab) on *RAS* conversion rate. In the group of 22 patients who received first-line chemotherapy alone, only 2 patients (9%) converted to *RAS* wild-type * in plasma, both 6 months after starting treatment (T2). In the remaining 20 patients (91%), *RAS* mutational status did not change in the follow up. Conversely, in the group of 50 patients who received first-line chemotherapy plus bevacizumab, 41 (82%) converted to *RAS* wild-type * in plasma; in the remaining 9 patients (18%), *RAS* mutational status did not change in the follow up. In the group of bevacizumab-treated patients, median PFS of patients who converted was significantly longer compared to that of patients who remained *RAS* mutant (9.3 vs. 5.9 months, *p* = 0.001) (Figure 1).

The logistic regression performed to address the relationship between *RAS* status conversion and the main covariates (sex, age, sidedness, type of mutation, and first-line chemotherapy with bevacizumab) indicated that the only independent variable significantly associated to RAS status conversion was the use of bevacizumab, with an OR = 45 95% CI (9–230).

## 4. Discussion

Several lines of evidence suggest that serial liquid biopsies are an excellent tool to monitor temporal heterogeneity in metastatic colorectal cancer over the course of treatments [15,16,17]. To date, liquid biopsies have shown the selective pressure of anti-EGFR therapies in patients with RAS-wild-type * colorectal tumors, in that acquired resistance to EGFR blockade is often driven by the emergence of *KRAS/NRAS* mutations in plasma [18]. More recently, we and others have reported that in *RAS* mutant mCRC, the conversion to *RAS* wild-type * status in plasma is a frequent event, ranging from 8% to 70% of cases according to studies [19,20,21,22,23], supporting that the evolutionary landscape of mCRC can lead to an unexpected negative selection of *RAS* mutant clones. Nevertheless, whether this conversion might depend on the evolutionary pressure induced by anti-VEGF treatments is still under debate. The aim of the present study was to investigate whether the phenomenon of *RAS* mutation conversion in colorectal cancer patients might be drug-dependent. For this purpose, we analyzed, through serial liquid biopsies, the impact of treatment regimen (CT alone vs. CT plus bevacizumab) on *RAS* conversion rate in a comprehensive population of patients with originally *RAS* mutant mCRC. Through the comparison between bevacizumab treated vs. untreated groups, our study demonstrates for the first time that the use of bevacizumab in the first-line treatment is the most significant factor for *RAS* conversion in plasma. This conversion occurred at different timepoints independently of sex, metastatic site, tumor sidedness, type of mutation, and clinical response. Our results obtained through serial liquid biopsies are consistent with the report by Epistolio et al., who recently characterized the primary tumor and paired liver metastases in 28 RAS mutant mCRCs, demonstrating that most mCRC patients treated with bevacizumab-containing regimens, but not those treated with CT alone, experienced a strong reduction of *RAS* mutant cells in liver metastasis, compared to the primary tumors resected before systemic therapy [24]. Accordingly, some studies performed with liquid biopsy support the hypothesis that anti-angiogenic therapy itself might induce changes in *RAS* mutational status in patients with mCRC monitored through liquid biopsies. Sunakawa et al. reported a 76% conversion rate from *RAS* mutant to *RAS* wild-type * 8 weeks after treatment with chemotherapy plus bevacizumab [21]. Similarly, Raimondi et al. described the loss of *RAS* mutant clones in plasma of mCRC patients, all treated with anti-VEGF [4]. More recently, Garcia de Santiago et al. showed that the *RAS* mutation status had changed to wild-type * in 73.9% of originally *RAS* mutant mCRC treated with antiangiogenics [23]. Conversely, Klein-Scory et al. reported that in patients with initially *RAS* mutated mCRC, *RAS* mutations rapidly disappeared in liquid biopsy during first-line therapy, independent of type of chemotherapy and irrespective of anti-VEGF treatments [5]. Unfortunately, in all these studies, the small number of patients and the lack of a comparative group treated with chemotherapy alone did not allow any significant conclusion regarding the role of antiangiogenics to induce *RAS* mutation conversion. How bevacizumab is associated to the conversion of *RAS* mutational status is currently unclear. Nevertheless, it is not surprising, in the light of the fact that cancer evolution is always shaped by the selective pressures imposed by the microenvironment. In colorectal cancer specifically, *RAS* mutant and *RAS* wild-type * cells always coexist in a sort of balance within the same tumor microenvironment, competing for space and resources [25]. A specific selective pressure, which suddenly modifies the cancer microenvironment, can give an account of the rates at which *RAS* mutant and *RAS* wild-type * clones appear and go extinct during the course of the disease. While Epistolio et al. suggest that inflammation and neo-angiogenesis can be taken in consideration as speculative selective pressure mechanisms, we hypothesize that it might depend on the ability of bevacizumab to maximally increase oxidative stress. In fact, although RAS-driven tumors strongly rely on increased reactive oxygen species (ROS) production to maintain their transformed state, a massive intracellular ROS increase is inefficiently scavenged in *RAS* mutant cells, leading to their selective ferroptosis, a kind of oxidative death [26,27]. Thus, being RAS-driven cancers particularly committed to keeping ROS levels within certain limits to be never exceeded, a viable strategy to target RAS mutant cancers is to sensitize cells to exogenous ROS inducers, shifting the redox state so that cells can no longer appropriately respond to further oxidative stress. This sensitization, which may result from the increased demand on intrinsic ROS-scavenging systems, is the basis of many anticancer drugs’ mechanism of action, including bevacizumab [28]. From a clinical perspective, *RAS* conversion was not found to be related to clinical response, in that RAS mutations disappeared irrespective of patient’s response or progression at the time of RAS conversion. In this regard, we agree with Klein-Scory et al. in that the disappearance of *RAS* mutation in plasma does not reflect a reduction in tumor load and does not represent a marker for therapeutic response [5]. The confirmation of ctDNA presence in the large majority of our samples attests the real negative selection of *RAS* mutated clones, disregarding the hypothesis that the lack of detection of *RAS* mutation in plasma might depend on the lack of ctDNA shedding. The observation that 30% of our patients who converted remained *RAS* wild-type * at the time of progressive disease further confirms such hypothesis. Rather, we believe that the decrease in RAS mutant cells might reflect the efficacy of bevacizumab-induced hypoxia to specifically eradicate *RAS* mutant population, leading to an increased prevalence of wild-type * cells. Despite the lack of association between *RAS* conversion and tumor burden reduction, bevacizumab-treated patients who converted to *RAS* wild-type * in plasma had a significantly longer PFS compared to patients who remained *RAS* mutant (9.3 vs. 5.9 months). This is consistent with previous studies reporting that *RAS* mutation rate is a significant predictor of PFS in mCRC patients treated with bevacizumab-containing first-line regimens [29]. Our study might have important clinical implications. In fact, the conversion of *RAS* mutated primary tumors to *RAS* wild-type * could possibly lead to their sensitization to EGFR-targeted treatments, which were otherwise precluded. In our population specifically, 30% of patients (those who converted at the time of disease progression), could have been candidates for this treatment. In this regard, the efficacy of cetuximab-based chemotherapy in patients with initially *RAS* mutated metastatic colorectal cancer, who displayed no detectable ctDNA *RAS* mutation after a first line treatment failure was already demonstrated [4,9]. More recently, Bouchahda et al. reported a median progression free survival of 8.2 months in patients without detectable ctDNA *RAS* mutation treated with cetuximab, as compared to 3.5 months in the ctDNA mutated patients who were treated according to standard recommendations [11]. These results are encouraging, taking into consideration the low efficacy of antiangiogenic treatments in second-line treatment in patients with *RAS* mutant tumors [30,31].

## 5. Conclusions

In conclusion, the RAS conversion in plasma following antiangiogenic treatments is a further example of how the interaction between genes and environment can influence colon cancer evolution. How this might impact in clinical practice is currently under investigation in phase II clinical trials.

## Figures and Tables

**Table 1:** Patient’s characteristics.

**Age, years**	
Mean	67
Range	44–88
**Sex, n.(%)**	
Male	46 (64%)
Female	26 (36%)
**Line of therapy**	
1st	72 (100%)
**Treatment received**	
CT plus Bev CT alone	50 (69%) 22 (31%)
**Location of primary tumor**	
Left	48 (66%)
Right	24 (34%)
**Site of metastasis**	
Single organ	26 (36%)
Multi-organ	46 (64%)
Liver	62 (86%)
Peritoneum	12 (17%)
Lymph-node	10 (14%)
Lung	6 (8%)
**Histology**	
Adenocarcinoma	72 (100%)
**RAS tissue/plasma baseline**	
Mutated *KRAS* G12D *KRAS* G12V *KRAS* G12A *KRAS* G12R/S *KRAS* G12C *KRAS* Q61 *KRAS* A146T *KRAS* G13D *NRAS* G12D *NRAS* G12C *NRAS* Q61R *NRAS* A146T *BRAF* V600E	72(100%) 19 (26%) 16 (22%) 5 (7%) 2 (3%) 6 (8%) 5 (7%) 5 (7%) 7 (10%) 3 (4%) 1(1%) 1 (1%) 1(1%) 1(1%)

CT: chemotherapy; Bev: Bevacizumab.

**Table 2:** Timing of RAS conversion and ctDNA confirmation test.

Pt. N.	Bevacizumab	RAS Mutation Tissue/Plasma Baseline	Timing of RAS Conversion (Months)	ctDNA Confirmation Test	
1	no	*KRAS G12V*	3	NGS	*TP53 c.524G>A p.R175H*
5	yes	*KRAS G12V*	9	methylation	*MSC ITGA4 EYA4*
7	yes	*KRAS G12V*	9	NGS	*SMAD4 c.1522G>T p.G508C*
9	yes	*KRAS G12V*	3	NGS	*TP53 c.659A>G p.Y220C*
22	yes	*KRAS G12V*	3	NGS	*SMAD4 c.1522G>T p.G508C*
27	yes	*KRAS G12V*	9	NGS	*TP53: c.659A>G p.Y220C*
39	yes	*KRAS G12V*	6	NGS	*PIK3CA c.1625A>T p.E542V*
24	yes	*KRAS G12V*	3	none	
2	no	*NRAS G12C*	3	methylation	*MAP3K MSC ITGA4 EYA4*
8	yes	*KRAS G12C*	6	NGS	*TP53: c.527G>T p.C176F*
26	yes	*KRAS G12C*	3	methylation	*MAP3K EYA4*
33	yes	*KRAS G12C*	12	NGS	*PIK3CA: c.3062A>G p. Y1021C*
36	yes	*KRAS G12C*	6	methylation	*MAP3K MSC ITGA4 EYA4 GRIA4*
3	yes	*KRAS G12D*	6	NGS	*TP53 c.743G>A p.R248Q*
6	yes	*KRAS G12D*	3	NGS	*PTEN c.209+6T>C*
16	yes	*KRAS G12D*	3	NGS	TP53 c.743G>A p.R248Q
21	yes	*KRAS G12D*	6	NGS	*TP53 c.524G>A p.R175H*
23	yes	*KRAS G12D*	9	none	
28	yes	*KRAS G12D*	3	NGS	*PIK3CA: c.3062A>G p. Y1021C*
30	yes	*KRAS G12D*	3	NGS	*TP53 c.844C>T p.R282W*
37	yes	*KRAS G12D*	9	NGS	*TP53 c.517G>A p.V173M*
34	yes	*KRAS G12D*	6	none	
35	yes	*KRAS G12D*	9	NGS	*FBXW7 c.1513C>T p.R505C; AKT1 c.49G>A p.E17K*
32	yes	*KRAS G12D*	6	none	
38	yes	*KRAS G12D*	9	NGS	*TP53 c.524G>A p.R175H*
43	yes	*KRAS G12D*	12	NGS	*PIK3CA c.1625A>T p.E542V*
17	yes	*KRAS G12A*	6	methylation	*MAP3K MSC ITGA4 EYA4 GRIA4*
19	yes	*KRAS G12A*	6	NGS	*PIK3CA c.3140A>G p.H1047R*
41	yes	*KRAS G12A*	6	methylation	*EYA4 GRIA4*
14	yes	*KRAS G13D*	3	NGS	*MAP2K1 c.171G>T p.K57N*
20	yes	*KRAS G13D*	6	methylation	*MAP3K MSC ITGA4 EYA4 GRIA4*
4	yes	*KRAS A146t*	3	none	
25	yes	*KRAS A146T*	6	NGS	*TP53 c.817C>T p.T273C*
29	yes	*KRAS A146T*	9	methylation	*MAP3K ITGA4 EYA4 GRIA4*
42	yes	*KRAS A146T*	12	NGS	*DDR2 c.1376C>T p.S459F*
12	yes	*KRAS Q61K*	3	NGS	*TP53: c.659A>G p.Y220C*
13	yes	*KRAS Q61K*	6	NGS	*PIK3CA: c.3062A>G p. Y1021C*
40	yes	*KRAS Q61H*	12	NGS	*TP53 c.401T>C p.F134S*
11	yes	*NRAS G12D*	3	NGS	*FBXW7 c.1513C>T p.R505C*
10	yes	*NRAS G12D*	6	none	
15	yes	*NRAS G12D*	9	none	
18	yes	*NRAS A146T*	3	methylation	*ITGA4 EYA4*
31	yes	*NRAS Q61R*	3	NGS	*SMAD4 c.989A>C p.E330A*

Pt: patients; ctDNA: circulating tumor DNA; NGS: next generation sequencing.

**Table 3:** Dynamics of the RAS conversion at different timepoints.

Ras Mutation Tissue/Plasma Baseline	Timing of First Ras Conversion (Months)	Response at First Ras Conversion	Ras Status at Serial Timepoints (* PD)			
			3 Mo	6 Mo	9 Mo	12 Mo
KRAS G12V	3	SD	wild-type	wild-type	wild-type	wild-type *
KRAS G12V	9	PD	KRAS G12V	KRAS G12V	wild-type *	wild type
KRAS G12V	9	PD	KRAS G12V	KRAS G12V	wild-type *	wild-type
KRAS G12V	3	PR	wild-type	wild-type	wild-type	wild-type
KRAS G12V	3	PR	wild-type	wild-type	wild-type	wild-type *
KRAS G12V	3	PR	wild-type	wild-type	wild-type	wild-type
KRAS G12V	9	PR	KRAS G12V	KRAS G12V	wild-type	wild-type
KRAS G12V	6	PR	KRAS G12V	wild-type	wild-type	wild-type *
KRAS G12C	6	PR	KRAS G12C	wild-type	wild-type	wild-type *
KRAS G12C	3	PR	wild-type	wild-type	wild-type	wild-type *
KRAS G12C	6	PR	KRAS G12C	wild-type	wild-type	wild-type
KRAS G12C	12	PD	KRAS G12C	KRAS G12C	KRAS G12C	wild-type *
KRAS G12D	6	PD	KRAS G12D	wild-type *	wild-type	wild type
KRAS G12D	3	PR	wild-type	wild-type	wild-type	KRAS G12D*
KRAS G12D	6	SD	KRAS G12D	wild-type	wild-type *	wild-type
KRAS G12D	9	PD	KRAS G12D	KRAS G12D	wild-type *	wild-type
KRAS G12D	3	PR	wild-type	wild-type	wild-type	wild type *
KRAS G12D	6	PR	KRAS G12D	wild-type	wild-type	wild-type
KRAS G12D	3	SD	wild-type	wild-type	wild-type *	KRAS Q61H
KRAS G12D	6	SD	G12D	wild-type	wild-type	wild type *
KRAS G12D	9	PD	KRAS G12D	KRAS G12D	wild-type *	wild-type
KRAS G12D	9	PD	KRAS G12D	KRAS G12D	wild-type *	wild-type
KRAS G12D	9	SD	KRAS G12D	KRAS G12D	wild-type	wild type
KRAS G12D	12	PD	KRAS G12D	KRAS G12D	KRAS G12D	wild type *
KRAS G12D	3	SD	wild-type	wild-type	wild-type	KRAS Q61K*
KRAS G12A	6	SD	KRAS G12A	wild-type	wild-type *	wild-type
KRAS G12A	6	PR	KRAS G12A	wild-type	wild-type	wild-type
KRAS G12A	6	CR	KRAS G12A	wild-type	wild-type	wild-type
KRAS G13D	3	PR	wild-type	wild-type	wild-type	KRAS G13D *
KRAS G13D	6	PR	KRAS G13D	wild-type	wild-type	wild-type
KRAS A146T	3	PR	wild-type	wild-type	wild-type *	KRAS G12C
KRAS A146T	6	PD	KRAS A146	wild-type *	wild-type	wild-type
KRAS A146T	9	CR	KRAS A146T	KRAS A146T	wild-type	wild type
KRAS A146T	12	PR	KRASA146P/T/V	KRASA146P/T/V	KRASA146P/T/V	wild-type
KRAS Q61K	3	PR	wild-type	wild-type	wild-type	wild-type *
KRAS Q61K	6	SD	KRAS Q61K	wild-type	wild-type *	wild-type
KRAS Q61H	12	PD	KRAS Q61H	KRAS Q61H	KRAS Q61H	wild type *
NRAS G12C	3	PR	wild-type	wild-type	wild-type	wild-type *
NRAS G12D	6	PD	NRAS G12D	wild-type *	wild-type	wild-type
NRAS G12D	3	SD	wild-type	wild-type	wild-type*	wild type
NRAS G12D	9	PD	NRAS G12D	NRAS G12D	wild-type *	wild-type
NRAS A146T	3	SD	wild-type	wild-type *	wild-type	wild-type
NRAS Q61R	3	SD	wild-type	wild-type *	wild-type	wild-type

Mo: months; * PD: progressive disease; SD: stable disease; PR: partial response; CR: complete response.

## Data Availability

Data sharing is not applicable to this article.
